# Physical Activity Adherence Related to Body Composition and Physical Fitness in Spanish Older Adults: 8 Years-Longitudinal EXERNET-Study

**DOI:** 10.3389/fpsyg.2022.858312

**Published:** 2022-04-26

**Authors:** Fabio Jiménez-Zazo, Antonio Hernández-Martínez, Cristina Romero-Blanco, Esther Cabanillas, Jorge Pérez-Gómez, Ignacio Ara, José A. Casajús, Germán Vicente-Rodríguez, Eva Gesteiro, Marcela González-Gross, Susana Aznar

**Affiliations:** ^1^PAFS Research Group, Faculty of Sports Sciences, University of Castilla-La Mancha, Toledo, Spain; ^2^Exercise and Health in Special Population Spanish Research Net, Zaragoza, Spain; ^3^Department of Nursing, Faculty of Nursing, University of Castilla-La Mancha, Ciudad Real, Spain; ^4^PAFS Research Group, Faculty of Nursing, University of Castilla-La Mancha, Ciudad Real, Spain; ^5^HEME Research Group, University of Extremadura, Cáceres, Spain; ^6^GENUD Toledo Research Group, Faculty of Sports Sciences, University of Castilla-La Mancha, Toledo, Spain; ^7^Biomedical Research Networking Center on Frailty and Healthy Aging (CIBERFES), Madrid, Spain; ^8^Growth, Exercise, Nutrition and Development Research Group, IIS Aragón, Zaragoza, Spain; ^9^Department of Physiatry and Nursing, Faculty of Health Sciences, University of Zaragoza, Zaragoza, Spain; ^10^CITA, Agroalimentary Institute of Aragón, Universidad de Zaragoza, Zaragoza, Spain; ^11^Physiopathology of Obesity and Nutrition Networking Biomedical Research Center, Zaragoza, Spain; ^12^Department of Physiatry and Nursing, Faculty of Health and Sport Science, Huesca, Spain; ^13^ImFine Research Group, Department of Health and Human Performance, Universidad Politécnica de Madrid, Madrid, Spain

**Keywords:** biobehavioral sciences, transtheoretical model (TTM), stages of change (SoC), physical activity, older adult, body composition, physical fitness

## Abstract

**Introduction:**

The multiple benefits of physical activity (PA) compared to the current lack of adherence to this behavior in older people makes it necessary to identify the factors related to its long-term dropout, therefore, the aim of this study was: (i) to study the evolution of behavior change for PA through stages of change (SoC) after 8 years and, to identify the associated factors related to the worsening of SoC for PA.

**Methods:**

A total of 714 Spanish older adults (>65 years) participated in the Longitudinal elderly EXERNET study 8 years follow-up. SoC for PA from Transtheoretical model of behavior change (TTM), body composition [BMI, fat mass, lean mass, waist circumference (WC), and hip circumference (HC)] and physical fitness (EXERNET battery fitness tests) were recorded. A multivariate binary logistic regression model was used to identify the potential predictive factors of worsening SoC for PA.

**Results:**

Three hundred and sixty participants (50.4%) worsening their SoC for PA after 8 years. Predictors factors associated with worsening of SoC were: HC (OR = 1.022; 95% CI: 1.001, 1.044), Lower body strength (OR = 0.940; 95% CI: 0.887, 0.997) and aerobic endurance at the 6 min-walk test (OR = 0.997; 95% CI: 0.995, 0.999).

**Conclusion:**

This study highlights that we need to identify adults with high HC, lower body strength and lower aerobic endurance to design a tailored PA program. Identifying the determinants of losing adherence to PA will help in the creation, design, and evaluation of exercise programs with the goal of reducing older adults’ drop-outs rates.

## Introduction

The current rate of aging population will be a challenge for health systems in the coming years. In 2030, it is expected that older adults (≥60 years old) will represent 12% of the world’s population, this value will increase to 16% by 2050 (United Nations, 2019). In Spain, values above 25% are expected to be reached by 2033 (Instituto Nacional de Estadística, 2018). Physical activity (PA) plays a very important role in the prevention of chronic diseases in older adults (i.e., cardiovascular diseases, metabolic diseases, musculoskeletal disorders, and cancer) (Warburton and Bredin, 2017). Physical activity is a key part in any healthy aging lifestyle (Cartee et al., 2016). The World Health Organization (WHO) recommends that older adults should engage in 150–300 min/week of at least moderate-intensity activities and muscle strengthening activity twice weekly (Bull et al., 2020). Despite the many health benefits of regular PA, few older adults meet the recommended guidelines (Bauman et al., 2016).

Long periods of inactivity or sedentary lifestyle in older adults population lead to deconditioning which involves a main decrease in muscle mass, with the consequent decrease in strength/endurance and aerobic capacity (Kortebein, 2009). On the other hand, an age-related progressive decrease in muscle mass (Mitchell et al., 2012) and an gradual increase in body fat percentage has been observed (Imboden et al., 2017; Westerterp, 2018). This term has been named sarcopenic obesity (Choi, 2016). Older adults with sarcopenic obesity tend to a functional decline (Kohara, 2014) and this fact can lead to a lower adherence to PA programs. The good news are that regular PA and/or exercise have positive effects in older adults such as: increases muscle mass and muscle function (Beaudart et al., 2017; Westerterp, 2018), and consequently maintains or increases their physical fitness (Milanović et al., 2013) and helps to maintain functional capacity and independence (Mazzeo and Tanaka, 2001). Therefore, it is not only important to motivate older adults to start an exercise program but to make sure they maintain it. So, to understand why people dropout PA and exercise programs and the factors involved in this process, seems crucial to develop tailored interventions that are sustained over time for older adults, focused on PA and exercise.

Behavioral theories help us to understand and predict behavior change in the population (Chase, 2015; Gourlan et al., 2016). Theory-based interventions on PA behavior based on a single theory have shown to be more effective than those based on a combination of theories (Gourlan et al., 2016). One of the most widely used and successful theories/models in promoting PA behaviors is Transtheoretical Model of Change (TTM; Gourlan et al., 2016). Initially it was used in studies to tobacco cessation, but over time its use has extended to promote healthy habits including the practice of PA. A recent review (Jiménez-Zazo et al., 2020) has presented the benefits of using TTM for PA in older adults.

The TTM was proposed by Prochaska and DiClemente (1983), is composed mainly by four constructs: stages of change (SoC), processes of change, decisional balance and self-efficacy, this model assumes that behavior change is a dynamic process, which occurs through a temporal dimension in a sequence of stages and processes, by which the individual moves until reaching regular behavior (Prochaska and DiClemente, 1983). The SoC, are part of the core of this model. SoC are a descriptive construct that explain where the people are in their motivation to change and their current behavior change (Romain et al., 2018). The SoC are composed of five stages: Precontemplation, contemplation, preparation, action, and maintenance. The SoC categorize individuals from non-intentional to change (precontemplation) to the acquisition of a regular behavior after 6 months (maintenance) (Prochaska and DiClemente, 1983).

Therefore, understanding the SoC to regular PA in older adults and to relate it to their changes in body composition and physical fitness overtime can help to identify the factors that determine the drop out of regular PA in this population. Under this umbrella, the Spanish network of research in exercise and health for special populations (EXERNET), conducted the Longitudinal elderly EXERNET study, it was the first study measured functional fitness in independent non-institutionalized Spanish older adults population, from 2008–2009 to 2017–2018 (Gomez-Bruton et al., 2020) which also measured the SoC for PA. Therefore, our study aim was to study the evolution of behavior change for PA through SoC after 8 years and, to identify the associated factors related to the worsening of SoC for PA.

## Materials and Methods

### Study Sample

This longitudinal study was carried out from 2008–2009 to 2016–2017 on the framework of “Longitudinal elderly EXERNET study (EXERNET-Elder 3.0)” (Gomez-Bruton et al., 2020). This project included a representative sample of non-institutionalized Spanish older adults over 65 years. Older adults were recruited from six regions of Spain: Aragón, Castilla La Mancha, Castilla y León, Canarias, Extremadura, and Madrid. The exclusion criteria were: people under 60 years; those suffering from cancer and/or dementia; those who were living in nursing homes and/or were not independent or able to take care of themselves and those subjects using walking aids.

A total of 3,267 older adults participated in the study, but only 2,712 (*n* = 2086 female; 76.9%) in period T0 (2008–2009) and 827 (*n* = 622 female; 75.2%) in period T1 (2017–2018) completed a face-to-face interview about SoC for PA. The final sample consisted of 714 (*n* = 540 female; 75.6%) older adults, who participated in both assessment periods, they met the inclusion criteria and were included in the present study. Prior to participation in the trials, all subjects were informed of the objectives of the study, as well as its possible risks and benefits. Written informed consent was obtained from all participants. The study was performed according to the principles established with the Declaration of Helsinki (1964) as revised in 2000 in Edinburgh, and approved by the Research Ethics Committees of Aragón (Spain) (18/2008).

All participants were interviewed and evaluated by qualified-researchers, previously trained. The following variables were extracted from the project and considered for analysis: SoC for PA, anthropometrics values and physical fitness tests.

### Instruments

Socio-demographic characteristics about age and gender were collected through the structured questionnaire designed for the “EXERNET multi-center study” (Gomez-Cabello et al., 2011).

Physical activity SoC Questionnaire (Marcus et al., 1992) was used to study the process of adopting PA in older adults. The SoC in older adults has shown to be suitable for older adults and has a good internal and external validity for exercise behavior (Marshall and Biddle, 2001; Chase, 2015; Jiménez-Zazo et al., 2020), therefore, its use can contribute to improve the understanding of the PA-related behavior. The SoC consists of five major stages: Pre-contemplation (inactive, does not think about being active at all), Contemplation (inactive, but thinks about being active), Preparation (does some PA but it is not a regular behavior), Action (performs PA regularly but for a period of less than 6 months), and Maintenance (regular PA practice for more than 6 months) (Prochaska and DiClemente, 1983). Regular PA or exercise was described as those activities involving brisk walking, running, biking, swimming or any other activities where the exertion was at least as intense as these activities, and accumulated a minimum of 30 min or more of PA throughout the day, at least a frequency of 5 days/week.

Anthropometric measurements were measured with a TANITA BC-418, Tokyo, Japan. The data of body mass index (BMI) (kg/m^2^), fat mass (kg), lean mass (kg) were taken with light clothes and without shoes. Waist circumference (WC) was made with the subject standing upright, feet together and arms hanging freely at the sides, it was measured with a non-stretchable measuring tape at a midway level between lower edge of the rib cage and iliac crest. Hip circumference (HC) was measured as the maximum circumference around the buttocks posteriorly and the symphysis pubis anteriorly.

Physical fitness was evaluated through the “Senior Fitness Test” battery, together with a balance test and a walking speed test both specific for the older adults population. Static balance by “Flamingo test”: time in seconds that it is able to stand on one foot, up to a maximum of 60-s (Johnson and Nelson, 1969), lower body strength by the chair stand test: number of times you are able to stand up from a chair in 30-s (Rikli and Jones, 2013), upper body strength by arm curl test: number of times you perform a flexo-extension with a 2.5-kg or 4-kg dumbbell in 30-s (Rikli and Jones, 2013), lower body flexibility by the chair sit-and-reach test: distance in cm from the hands to the tips of the feet when flexing the trunk while sitting (Rikli and Jones, 2013), upper body flexibility by back scratch test: distance in cm between both hands when trying to touch each other diagonally from behind (Rikli and Jones, 2013), agility/dynamic balance by the 8-foot up-and-go test: time in seconds it takes to make a circuit starting from seating (Rikli and Jones, 2013), speed by the 30-m walk by brisk walking test: time in seconds it takes to walk 30-m (Carvalho et al., 2010) and aerobic endurance by the 6-min walk test: meters you are able to walk in 6-min (Rikli and Jones, 2013). All the tests were performed only once, except the one leg test, which was performed twice with each leg, the 8-foot up-and-go test and the 30-m walk test, which were also performed twice.

### Sample Size Calculation

The maximum modeling criterion for observational studies with multivariate analysis was used to calculate the sample size (Peduzzi et al., 1996). According to this criterion, a minimum of 10 events is required for each independent variable included in the original multivariate model. We considered the worsening of SoC during the period T0–T1 to be an event. Thus, if we include 17 independent variables in the original model, we require a minimum of 170 subjects with worsening SoC during the period under study. However, the research team chose to recruit the maximum number of participants to improve statistical power.

### Statistical Analysis

A descriptive statistical analysis using absolute and relative frequencies for qualitative variables and means and standard deviation (SD) for quantitative variables was performed. The main outcome variable was the worsening of SoC for PA after 8 years. The independent variables were: body composition (BMI, fat mass, lean mass, WC, and HC) and physical fitness (EXERNET battery fitness tests).

The analysis of potential predictive factors, which have been previously identified in the literature as risk factors of worsening SoC, was carried out in a bivariate analysis using Chi-square and Student’s *t*-test to estimate qualitative and quantitative variables, respectively, Adjusted odds ratios (aOR) were also estimated and its 95% confidence interval (IC 95%). Of these variables, and following Lemeshow’s statistical criteria, associations with *p*-values < 0.25 were selected for inclusion in the multivariate binary logistic regression model (Mickey and Greenland, 1989; Hosmer, Lemeshow and Rx, 2013), obtaining adjusted odds ratios (aOR) with their respective IC 95%. A *p* < 0.05 was considered significant. This model was built by using backward elimination (RV in SPSS). We used SPSS 20.0 (SPSS Inc., Chicago, IL, United States) for all statistical analyses.

## Results

The evolution of the SoC between periods 2008 and 2009 up to 2017 and 2018 together with the relationship between body composition and physical fitness (at baseline) with worsening SoC, are presented in Tables 1, 2.

**TABLE 1:** Distribution of SoC for PA between the periods T0 and T1.

	Period T0 (n, %)	Period T1 (n, %)
Precontemplation	166 (6.1%)	286 (34.6%)
Contemplation	108 (4.0%)	44 (5.3%)
Preparation	536 (19.8%)	109 (13.2%)
Action	101 (3.7%)	6 (0.7%)
Maintenance	1801 (66.4%)	382 (46.2%)

*Period T0, 2008–2009; Period T1, 2017–2018.*

**TABLE 2:** Evolution of the SoC for PA between the periods T0 and T1.

	Evolution of SoC for PA (n, %)
Worsening	360 (50.4%)
No change	282 (39.5%)
Improve	72 (10.1%)

*SoC, Stages of change; PA, Physical activity. Period T0, 2008–2009; Period T1, 2017–2018.*

### Identification and Evolution of Stages of Change Between Periods T0 and T1

Table 1, shows the distribution of the SoC for PA in older adults between the assessment periods T0 (2008–2009) and T1 (2017–2018) for the total number of participants in each assessment period T0 (*n* = 2712) and T1 (*n* = 827). There was an increase in the percentage distribution of older adults located in inactive stages (i.e., Precontemplation) of period T1 with respect to period T0.

In Table 2, we show the evolution of SoC over time only for those participants that completed a face-to-face interview about SoC for PA in both assessment periods (*n* = 714). The results highlighted that about 50% (*n* = 360) of the participants worsened their motivation for regular PA between the periods T0 and T1.

This article focused on the “worsening older adults,” i.e., those who went from more active SoC (period T0) to more inactive ones (period T1) (*n* = 360), and we analyzed the physical and environmental determinants, compare to those ones who did not change or improved their SoC (*n* = 354).

### Relationship Between Socio-Demographic Characteristics and Worsening Stages of Change: Bivariate Analysis

Significant differences was found in age (*p* = 0.009), but not in gender (*p* > 0.05; Table 3) with respect to the worsening of the SoC between the periods T0 and T1.

**TABLE 3:** Socio-demographic characteristics and SoC worsening between periods T0 and T1: Bivariate analysis.

Variable		SoC worsening periods T0–T1		
		No (*n* = 354)	Yes (*n* = 360)	*p*-value
Age	Mean (SD)	70.2 (4.39)	71.1 (4.81)	0.009
Gender				0.178
Woman		260 (48.1%)	280 (51.9%)	
Man		94 (54.0%)	80 (46.0%)	

*SoC, Stages of change; Period T0, 2008–2009; Period T1, 2017–2018; SD, standard deviation; Statistical significance: p < 0.05.*

### Relationship Between Body Composition and Worsening Stages of Change: Bivariate Analysis

Those older adults who worsened their SoC, at baseline (T0), registered significantly worse scores in BMI, WC, HC, and total fat mass (all, *p* < 0.05), that is, those older adults with higher: BMI, WC, HC, and total fat mass, tended to worsen their SoC over time, see Table 4. No significant differences in lean mass were found.

**TABLE 4:** Body composition and SoC worsening between periods T0 and T1: Bivariate analysis.

Variable		SoC worsening periods T0–T1		
		No (*n* = 354)	Yes (*n* = 360)	*p*-value
BMI	Mean (SD)	28.4 (3.62)	29.4 (3.99)	0.001
Waist circumference (cm)		91.2 (11.78)	93.8 (12.04)	0.003
Hip circumference (cm)		102.4 (7.80)	104.8 (8.95)	<0.001
Total Fat Mass (Kg)		25.5 (6.40)	26.6 (7.06)	0.023
Total Lean Mass (Kg)		44.6 (8.26)	44.4 (7.72)	0.692

*SoC, Stages of change; Period T0, 2008–2009; Period T1, 2017–2018; SD, standard deviation; BMI, Body mass index; Statistical significance: p < 0.05.*

### Relationship Between Physical Fitness and Worsening Stages of Change: Bivariate Analysis

The scores of balance, lower body strength, upper strength right and left, left lower flexibility, agility/dynamic balance, 30-m walk test and aerobic endurance were significantly worse at baseline (all, *p* < 0.05) for older adults who worsened their SoC in comparison with those who did not. No significant differences in upper flexibility and right lower flexibility were found (*p* > 0.05; Table 5).

**TABLE 5:** Physical fitness and SoC worsening between periods T0 and T1: Bivariate analysis.

Variable	SoC worsening periods T0–T1		
	No (*n* = 354)	Yes (*n* = 360)	*p*-value
Balance (s)	35.0 (21.12)	29.9 (21.70)	0.002
Lower body strength (rep)	15.4 (3.25)	14.4 (2.98)	<0.001
Right upper strength (rep)	17.8 (3.66)	16.8 (3.29)	<0.001
Left upper strength (rep)	17.9 (3.76)	17.0 (3.36)	0.001
Right lower flexibility (cm)	−2.3 (10.96)	−3.5 (9.80)	0.108
Left lower flexibility (cm)	−1.6 (11.23)	−3.3 (10.12)	0.038
Right upper flexibility (cm)	−6.7 (10.95)	−7.8 (11.24)	0.164
Left upper flexibility (cm)	−11.0 (10.68)	−12.0 (10.34)	0.202
Agility/dynamic balance (s)	5.3 (1.00)	5.5 (1.08)	0.001
30-m walk test (s)	15.9 (2.60)	17.0 (3.36)	<0.001
Aerobic endurance (m)	565.9 (84.09)	535.8 (79.80)	<0.001

*SoC, Stages of change; Period T0, 2008–2009; Period T1, 2017–2018; Statistical significance: p < 0.05.*

### Factors Associated With the Worsening of Stages of Change for Regular Physical Activity

After the bivariate analysis, a multivariate analysis was performed using binary logistic regression. The initial model included all potential predictors with *p*-values < 0.25 from Tables 3, 4. From all these factors, the final model comprised three predictors. Among the risk of worsening predictors, the following factors were identified: HC, lower body strength (rep) and aerobic endurance (m). Specifically, having greater HC was considered a risk factor while the protective factors were greater leg strength and greater aerobic endurance at the 6 min-walk test (Table 6).

**TABLE 6:** Factors associated with the worsening of the SoC between the T0 and T1 periods: Multivariate analysis.

Variables	SoC worsening periods T0–T1		
	*Coef*.*B*	*p*-value	aOR of worsening (95% CI)
Hip circumference (cm)	0.022	0.039	1.022 (1.001, 1.044)
Lower body strength (rep)	–0.062	0.039	0.940 (0.887, 0.997)
Aerobic endurance (m)	–0.003	0.016	0.997 (0.995, 0.999)
Cte	0.208		

*SoC, Stages of change; T0, 2008–2009; T1, 2017–2018; OR, Odd Ratio; aOR, Adjusted odd ratio; CI, 95% Confidence interval; Statistical significance: p < 0.05. The selected model has been developed using the SPSS backward stepwise procedure. Cte refers to the value of the constant of the multivariate analysis.*

## Discussion

This article has identified the correlates of decreasing PA practice in a sample of 714 Spanish older adults (>65 years) in a 8-years follow-up period (2008–2009 to 2017–2018). A higher HC, lower fitness levels in lower body strength and aerobic endurance at the 6 min-walk test at baseline, were related to decreasing PA and becoming sedentary in Spanish older adults after 8 years.

The study has used the SoC to identify the status of active versus non-active behavior in older adults. The popularity of the SoC lays on the possibility of tailoring interventions to individuals undergoing different SoC. This study takes a former step to determine how physical fitness and anthropometric variables can predict the change from active to inactive stages after a period of 8 years. Identifying the determinants of the worsening process of SoC for PA, will help to focus on tailored intervention exercise programs with the goal of reducing older adults’ drop-outs rates.

Our results have shown as a high HC at the baseline was correlated to decreasing PA practice in Spanish older adults after a period of 8 years. Changes in body composition, such as increased body fat and losses in skeletal muscle mass represent important risk factors for the incidence of cardiometabolic diseases such as hypertension, dyslipidemia, and type II diabetes mellitus in older adults (Sillanpää et al., 2008). In Spain, EXERNET multi-center study (Gomez-Cabello et al., 2011) found that more than 84% of older adults over 65 years had problems with their body weight (46.7% overweight and 36.7% obese), the correlation between increasing fat mass and decreasing PA in the older adults, is like a snake biting its own tail. An old person who increases body fat can drop PA and dropping PA can lead to an increase in percentage of body fat. More efforts are needed to control diet (calorie ingestion control) and exercise (caloric expenditure control) interventions, that target the maintenance and improvements of older adults anthropometric and body composition.

In regard to physical fitness, lower body strength and aerobic endurance, have been identified as determinant for decrease PA practice in older adults. It has been reported that the process of aging dramatically impacts functional abilities, such as sit-to-stand, gait, and stair climbing abilities (Reid and Fielding, 2012; Müller et al., 2021), mainly related to muscle power declines observed during the aging process (Izquierdo et al., 1999; Müller et al., 2021). Our results have identified older adults’ lower results in the chair-stand test and lower distance in the aerobic endurance 6-min walk test, as predictors to a decrease in their PA practice over a period of 8 years. Lower limb muscle power decreased progressively with age in both men and women (Alcazar et al., 2020), which leads to a decrease in muscle function, affecting to physical function and gait speed (Suetta et al., 2019). Resistance training can increase range of motion of a number of joints of inactive older individuals possibly due to an improvement in muscle strength (Fatouros et al., 2002). Our results show that there are no differences in upper body flexibility and right lower flexibility between older adults who worsened their SoC for PA and the ones who did not. Future exercise programs should focus on improving functional capacity by promoting lower limb strength work with the aim of achieving maintenance and independence in basic activities of daily living in older adults.

Older adults exercise programs that focus on lower limb and all body strength training in general, are important to promote adherence to PA (Kathleen and Adrienne, 2001). Moreover, a benefit in muscle mass will make older adults more efficient metabolically during given activities of daily living and therefore improving endurance capacity to the same given effort, i.e., walking faster.

Currently, there are many exercise programs for the older adults (Cadore et al., 2013), recently the VIVIFRAIL Project^1^ as a multicomponent individualized exercise program recognized by the Ministry of Health in Spain, includes progressive cardiovascular, resistance exercise and balance with the aim of improving the functional capacity of older people, i.e., improvement of the rate of falls, gait ability, balance, and strength performance (Izquierdo, 2019). It seems necessary to implement these kinds of programs in society to diminish PA practice decline, although it could be a much interesting option the supervised and individualized exercise programs as Eelder-fit to maximize training benefits in pre- and frail older adults (Moradell et al., 2020).

Older adults group (>65 years) is a very heterogeneous group, so it may have specific characteristics that require different strategies from other age groups. Within our research we have been able to identify the most relevant and predictive aspects related to the worsening of behavior change referred to the practice of regular PA in this group. Exercise programs that are focused on favorable changes in anthropometric and body composition, increasing fitness levels in lower body strength and aerobic endurance are encouraged.

## Limitations

Our study has different limitations, the sample only includes independent non-institutionalized older adults, generally from civic and sports centers, so the results must be interpreted with caution, since it may be a bias for the extrapolation of these results to other groups of older population. This study did not take into account other factors which could lead to a dropout from PA in older adults as age, race (Caucasian), poorer level of physical function, being physically less active, lack of time, family reconciliation, health problems, low levels of motivation and self-efficacy, perceived barriers, lack of knowledge about the benefits of PA, lack of social support, weather, as well as the environment and the possibilities it generates to be physically active (Resnick and Spellbring, 2000; Schutzer and Graves, 2004; O’Shea et al., 2007; Gillette et al., 2015; Kassavou et al., 2015; Geirsdottir et al., 2017). However, we have several strengths, firstly, this is a prospective 8-year follow-up study with a good sample size (*n* = 714), the age of the participants (>65 years), and the variability of the tests carried out within the study.

## Conclusion

In a 8-year follow-up (2008–2009 to 2017–2018) study of 714 independent non-institutionalized older adults, the worsening of SoC for PA was associated with high HC, low body strength and low aerobic endurance at baseline. This study highlights that we need to identify adults with high HC, lower body strength and lower aerobic endurance to design a tailored PA program. Identifying the determinants of losing adherence to PA will help in the creation, design, and evaluation of exercise programs with the goal of reducing older adults’ drop-outs rates. Exercise programs that are focused on favorable changes in anthropometric and body composition, increasing fitness levels in lower body strength and aerobic endurance are encouraged.

## Data Availability Statement

The raw data supporting the conclusions of this article will be made available by the authors, without undue reservation.

## Ethics Statement

The studies involving human participants were reviewed and approved by the Research Ethics Committees of Aragón (Spain) (18/2008). The patients/participants provided their written informed consent to participate in this study.

## Author Contributions

FJ-Z and SA: conceptualization and writing – original draft preparation. JP-G, IA, JC, GV-R, MG-G, and SA: methodology and project administration. FJ-Z, AH-M, and SA: formal analysis and statistical analysis. FJ-Z, CR-B, EC, JP-G, IA, JC, GV-R, EG, MG-G, and SA: investigation and data curation. FJ-Z, CR-B, EC, JP-G, IA, JC, GV-R, MG-G, and SA: resources. FJ-Z, AH-M, CR-B, EC, JP-G, IA, JC, GV-R, EG, MG-G, and SA: writing – review and editing, visualization, and supervision. FJ-Z, JP-G, IA, JC, GV-R, MG-G, and SA: funding acquisition. All authors have read and approved the final version of the manuscript, and agreed with the order of presentation of the authors.

## Conflict of Interest

The authors declare that the research was conducted in the absence of any commercial or financial relationships that could be construed as a potential conflict of interest.

## Publisher’s Note

All claims expressed in this article are solely those of the authors and do not necessarily represent those of their affiliated organizations, or those of the publisher, the editors and the reviewers. Any product that may be evaluated in this article, or claim that may be made by its manufacturer, is not guaranteed or endorsed by the publisher.

## Footnotes

1
www.vivifrail.com

## Abbreviations

PA: Physical activity
SoC: Stages of change
BMI: Body mass index
TTM: Transtheoretical model of change
WC: Waist circumference
HC: Hip circumference.

## References

[B1] Alcazar J. Aagaard P. Haddock B. Kamper R. S. Hansen S. K. Prescott E. (2020). Age- and sex-specific changes in lower-limb muscle power throughout the lifespan. *J. Gerontol. A Biol. Sci. Med. Sci.* 75 1369–1378. 10.1093/gerona/glaa013 31943003

[B2] Bauman A. Merom D. Bull F. C. Buchner D. M. Fiatarone Singh M. A. (2016). Updating the evidence for physical activity: summative reviews of the epidemiological evidence, prevalence, and interventions to promote “active aging”. *Gerontologist* 56(Suppl. 2) S268–S280. 10.1093/geront/gnw031 26994266

[B3] Beaudart C. Dawson A. Shaw S. C. Harvey N. C. Kanis J. A. Binkley N. (2017). Nutrition and physical activity in the prevention and treatment of sarcopenia: systematic review. *Osteoporos. Int.* 28 1817–1833. 10.1007/s00198-017-3980-9 28251287 PMC5457808

[B4] Bull F. C. Al-Ansari S. S. Biddle S. Borodulin K. Buman M. P. Cardon G. (2020). World health organization 2020 guidelines on physical activity and sedentary behaviour. *Br. J. Sports Med.* 54 1451–1462. 10.1136/bjsports-2020-102955 33239350 PMC7719906

[B5] Cadore E. L. Rodríguez-Mañas L. Sinclair A. Izquierdo M. (2013). Effects of different exercise interventions on risk of falls, gait ability, and balance in physically frail older adults: a systematic review. *Rejuvenation Res.* 16 105–114. 10.1089/rej.2012.1397 23327448 PMC3634155

[B6] Cartee G. D. Hepple R. T. Bamman M. M. Zierath J. R. (2016). Exercise promotes healthy aging of skeletal muscle. *Cell Metab.* 23 1034–1047. 10.1016/j.cmet.2016.05.007 27304505 PMC5045036

[B7] Carvalho C. Sunnerhagen K. S. Willén C. (2010). Walking speed and distance in different environments of subjects in the later stage post-stroke. *Physiother. Theory Pract.* 26 519–527. 10.3109/09593980903585042 20649494

[B8] Chase J. A. (2015). Interventions to increase physical activity among older adults: a meta-analysis. *Gerontologist* 55 706–718. 10.1093/geront/gnu090 25298530 PMC4542588

[B9] Choi K. M. (2016). Sarcopenia and sarcopenic obesity. *Korean J. Intern. Med.* 31 1054–1060. 27809450 10.3904/kjim.2016.193 PMC5094937

[B10] Fatouros I. G. Taxildaris K. Tokmakidis S. P. Kalapotharakos V. Aggelousis N. Athanasopoulos S. (2002). The effects of strength training, cardiovascular training and their combination on flexibility of inactive older adults. *Int. J. Sports Med.* 23 112–119. 10.1055/s-2002-20130 11842358

[B11] Geirsdottir O. G. Chang M. Briem K. Jonsson P. V. Thorsdottir I. Ramel A. (2017). Gender, success, and drop-out during a resistance exercise program in community dwelling old adults. *J. Aging Res.* 2017:5841083. 10.1155/2017/5841083 28890833 PMC5584358

[B12] Gillette D. B. Petrescu-Prahova M. Herting J. R. Belza B. (2015). A pilot study of determinants of ongoing participation in EnhanceFitness: a community-based group exercise program for older adults. *J. Geriatr. Phys. Ther.* 38 194–201. 10.1519/JPT.0000000000000041 25695473 PMC4540700

[B13] Gomez-Bruton A. Navarrete-Villanueva D. Pérez-Gómez J. Vila-Maldonado S. Gesteiro E. Gusi N. (2020). The effects of age, organized physical activity and sedentarism on fitness in older adults: an 8-year longitudinal study. *Int. J. Environ. Res. Public Health* 17:4312. 10.3390/ijerph17124312 32560257 PMC7345727

[B14] Gomez-Cabello A. Pedrero-Chamizo R. Olivares P. R. Luzardo L. Juez-Bengoechea A. Mata E. (2011). Prevalence of overweight and obesity in non-institutionalized people aged 65 or over from Spain: the elderly EXERNET multi-centre study. *Obes. Rev.* 12 583–592. 10.1111/j.1467-789X.2011.00878.x 21535360

[B15] Gourlan M. Bernard P. Bortolon C. Romain A. J. Lareyre O. Carayol M. (2016). Efficacy of theory-based interventions to promote physical activity. A meta-analysis of randomised controlled trials. *Health Psychol. Rev.* 10 50–66. 10.1080/17437199.2014.981777 25402606

[B16] Hosmer D. W. Lemeshow S. Rx S. (2013). “Model-building strategies and methods for logistic regression,” in *Applied Logistic Regression*, 3rd Edn, ed. Wiley (Hoboken, NJ: John Wiley & Sons).

[B17] Imboden M. T. Welch W. A. Swartz A. M. Montoye A. H. Finch H. W. Harber M. P. (2017). Reference standards for body fat measures using GE dual energy x-ray absorptiometry in Caucasian adults. *PLoS One* 12:e0175110. 10.1371/journal.pone.0175110 28388669 PMC5384668

[B18] Instituto Nacional de Estadística (2018). *Proyecciones de Población 2018 [Online]. Instituto Nacional de Estadísitca.* Available online at: https://www.ine.es/prensa/pp_2018_2068.pdf (accessed 17.12.2019)

[B19] Izquierdo M. (2019). [Multicomponent physical exercise program: Vivifrail]. *Nutr. Hosp.* 36 50–56. 31189323 10.20960/nh.02680

[B20] Izquierdo M. Ibañez J. Gorostiaga E. Garrues M. Zúñiga A. Antón A. (1999). Maximal strength and power characteristics in isometric and dynamic actions of the upper and lower extremities in middle-aged and older men. *Acta Physiol. Scand.* 167 57–68. 10.1046/j.1365-201x.1999.00590.x 10519978

[B21] Jiménez-Zazo F. Romero-Blanco C. Castro-Lemus N. Dorado-Suárez A. Aznar S. (2020). Transtheoretical model for physical activity in older adults: systematic review. *Int. J. Environ. Res. Public Health* 17:9262. 10.3390/ijerph17249262 33322327 PMC7763623

[B22] Johnson B. L. Nelson J. K. (1969). *Practical Measurements for Evaluation in Physical Education.* Minneapolis, MN: Burgess Publishing Company.

[B23] Kassavou A. Turner A. French D. P. (2015). The role of walkers’ needs and expectations in supporting maintenance of attendance at walking groups: a longitudinal multi-perspective study of walkers and walk group leaders. *PLoS One* 10:e0118754. 10.1371/journal.pone.0118754 25774527 PMC4361621

[B24] Kathleen A. M. Adrienne R. S. (2001). Who will stay and who will go? A review of older adults’ adherence to randomized controlled trials of exercise. *J. Aging Phys. Act.* 9 91–114. 10.1123/japa.9.2.91

[B25] Kohara K. (2014). Sarcopenic obesity in aging population: current status and future directions for research. *Endocrine* 45 15–25. 10.1007/s12020-013-9992-0 23821364

[B26] Kortebein P. (2009). Rehabilitation for hospital-associated deconditioning. *Am. J. Phys. Med. Rehabil.* 88 66–77. 10.1097/phm.0b013e3181838f70 18688198

[B27] Marcus B. H. Rossi J. S. Selby V. C. Niaura R. S. Abrams D. B. (1992). The stages and processes of exercise adoption and maintenance in a worksite sample. *Health Psychol.* 11 386–395. 10.1037//0278-6133.11.6.386 1286658

[B28] Marshall S. J. Biddle S. J. (2001). The transtheoretical model of behavior change: a meta-analysis of applications to physical activity and exercise. *Ann. Behav. Med.* 23 229–246. 10.1207/S15324796ABM2304_2 11761340

[B29] Mazzeo R. S. Tanaka H. (2001). Exercise prescription for the elderly: current recommendations. *Sports Med.* 31 809–818. 10.2165/00007256-200131110-00003 11583105

[B30] Mickey R. M. Greenland S. (1989). The impact of confounder selection criteria on effect estimation. *Am. J. Epidemiol.* 129 125–137. 10.1093/oxfordjournals.aje.a115101 2910056

[B31] Milanoviæ Z. Pantelić S. Trajkoviæ N. Sporiš G. Kostiæ R. James N. (2013). Age-related decrease in physical activity and functional fitness among elderly men and women. *Clin. Interv. Aging* 8 549–556. 10.2147/CIA.S44112 23723694 PMC3665513

[B32] Mitchell W. K. Williams J. Atherton P. Larvin M. Lund J. Narici M. (2012). Sarcopenia, dynapenia, and the impact of advancing age on human skeletal muscle size and strength; a quantitative review. *Front. Physiol.* 3:260. 10.3389/fphys.2012.00260 22934016 PMC3429036

[B33] Moradell A. Navarrete-Villanueva D. Fernández-García ÁI. Sagarra-Romero L. Marín-Puyalto J. Pérez-Gómez J. (2020). Effects of a multicomponent exercise program, a detraining period and dietary intake prediction of body composition of frail and pre-frail older adults from the EXERNET elder 3.0 study. *Sustainability* 12:9894. 10.3390/su12239894

[B34] Müller D. C. Boeno F. P. Izquierdo M. Aagaard P. Teodoro J. L. Grazioli R. (2021). Effects of high-intensity interval training combined with traditional strength or power training on functionality and physical fitness in healthy older men: a randomized controlled trial. *Exp. Gerontol.* 149:111321. 10.1016/j.exger.2021.111321 33757813

[B35] O’Shea S. D. Taylor N. F. Paratz J. D. (2007). …But watch out for the weather: factors affecting adherence to progressive resistance exercise for persons with COPD. *J. Cardiopulm. Rehabil. Prev.* 27 166–74;quiz175–6. 10.1097/01.HCR.0000270686.78763.c8 17558200

[B36] Peduzzi P. Concato J. Kemper E. Holford T. R. Feinstein A. R. (1996). A simulation study of the number of events per variable in logistic regression analysis. *J. Clin. Epidemiol.* 49 1373–1379. 10.1016/s0895-4356(96)00236-3 8970487

[B37] Prochaska J. O. DiClemente C. C. (1983). Stages and processes of self-change of smoking: toward an integrative model of change. *J. Consult. Clin. Psychol.* 51 390–395. 10.1037/0022-006x.51.3.390 6863699

[B38] Reid K. F. Fielding R. A. (2012). Skeletal muscle power: a critical determinant of physical functioning in older adults. *Exerc. Sport Sci. Rev.* 40 4–12. 10.1097/JES.0b013e31823b5f13 22016147 PMC3245773

[B39] Resnick B. Spellbring A. M. (2000). Understanding what motivates older adults to exercise. *J. Gerontol. Nurs.* 26 34–42. 10.3928/0098-9134-20000301-08 11111629

[B40] Rikli R. E. Jones C. J. (2013). *Senior Fitness test Manual.* Champaign, IL: Human kinetics.

[B41] Romain A. J. Caudroit J. Hokayem M. Bernard P. (2018). Is there something beyond stages of change in the transtheoretical model? The state of art for physical activity. *Can. J. Behav. Sci.* 50 42–53. 10.1037/cbs0000093

[B42] Schutzer K. A. Graves B. S. (2004). Barriers and motivations to exercise in older adults. *Prev. Med.* 39 1056–1061. 10.1016/j.ypmed.2004.04.003 15475041

[B43] Sillanpää E. Häkkinen A. Nyman K. Mattila M. Cheng S. Karavirta L. (2008). Body composition and fitness during strength and/or endurance training in older men. *Med. Sci. Sports Exerc.* 40 950–958. 10.1249/MSS.0b013e318165c854 18408601

[B44] Suetta C. Haddock B. Alcazar J. Noerst T. Hansen O. M. Ludvig H. (2019). The Copenhagen Sarcopenia Study: lean mass, strength, power, and physical function in a Danish cohort aged 20-93 years. *J. Cachexia Sarcopenia Muscle* 10 1316–1329. 10.1002/jcsm.12477 31419087 PMC6903448

[B45] United Nations (2019). Selected results of the 2019 UN world population projections. *Popul. and Dev. Rev.* 45 689–694. 10.1111/padr.12288

[B46] Warburton D. E. R. Bredin S. S. D. (2017). Health benefits of physical activity: a systematic review of current systematic reviews. *Curr. Opin. Cardiol.* 32 541–556. 10.1097/HCO.0000000000000437 28708630

[B47] Westerterp K. R. (2018). Changes in physical activity over the lifespan: impact on body composition and sarcopenic obesity. *Obes. Rev.* 19(Suppl. 1) 8–13. 10.1111/obr.12781 30511504

